# Do tuition-free lower secondary education policies matter for antenatal care among women in sub-saharan African countries?

**DOI:** 10.1186/s12884-024-06406-1

**Published:** 2024-04-08

**Authors:** Bijetri Bose, Amy Raub, Aleta Sprague, Alfredo Martin, Pragya Bhuwania, Rachel Kidman, Jody Heymann

**Affiliations:** 1 WORLD Policy Analysis Center, Fielding School of Public Health, University of California, Los Angeles, USA; 2 Department of Family, Population and Preventive Medicine, Stony Brook University, Stony Brook, New York USA

**Keywords:** Tuition-free secondary education, Tuition-free primary education, Antenatal care, Reproductive health, Sub-Saharan Africa, SDGs

## Abstract

**Background:**

Antenatal care (ANC) is critical to reducing maternal and infant mortality. However, sub-Saharan Africa (SSA) continues to have among the lowest levels of ANC receipt globally, with half of mothers not meeting the WHO minimum recommendation of at least four visits. Increasing ANC coverage will require not only directly reducing geographic and financial barriers to care but also addressing the social determinants of health that shape access. Among those with the greatest potential for impact is maternal education: past research has documented a relationship between higher educational attainment and antenatal healthcare access, as well as related outcomes like health literacy and autonomy in health decision-making. Yet little causal evidence exists about whether changing educational policies can improve ANC coverage. This study fills this research gap by investigating the impact of national-level policies that eliminate tuition fees for lower secondary education in SSA on the number of ANC visits.

**Methods:**

To estimate the effect of women’s exposure to tuition-free education policies at the primary and lower secondary levels on their ANC visits, a difference-in-difference methodology was employed. This analysis leverages the variation in the timing of education policies across nine SSA countries.

**Results:**

Exposure to tuition-free primary and lower secondary education is associated with improvements in the number of ANC visits, increasing the share of women meeting the WHO recommendation of at least four ANC visits by 6–14%. Moreover, the impact of both education policies combined is greater than that of tuition-free primary education alone. However, the effects vary across individual treatment countries, suggesting the need for further investigation into country-specific dynamics.

**Conclusions:**

The findings of this study have significant implications for policymakers and stakeholders seeking to improve ANC coverage. Removing the tuition barrier at the secondary level has shown to be a powerful strategy for advancing health outcomes and educational attainment. As governments across Africa consider eliminating tuition fees at the secondary level, this study provides valuable evidence about the impacts on reproductive health outcomes. While investing in free education requires initial investment, the long-term benefits for both human development and economic growth far outweigh the costs.

**Supplementary Information:**

The online version contains supplementary material available at 10.1186/s12884-024-06406-1.

## Introduction

Antenatal care (ANC) services are crucial to improving overall reproductive health and reducing maternal and infant mortality [1–3]. These services encompass various practices, such as nutritional counseling, screening for infections, provision of iron and folic acid supplements, immunizations, and identification and treatment of high-risk conditions like HIV, malaria, and eclampsia/preeclampsia [4]. In sub-Saharan Africa (SSA), even a single ANC visit can reduce the risk of neonatal mortality by 39% [5]. ANC visits also increase the likelihood of having skilled birth attendants during delivery, which has been identified as one of the most effective strategies for lowering maternal mortality risks [6, 7].

Nevertheless, while between 76% and 87% of women in SSA receive at least one ANC visit [8, 9], only around half are meeting the World Health Organization (WHO) recommendation of at least four visits [10, 11], with vast differences across countries [8, 12]. Meanwhile, maternal mortality in the region stands at 545 deaths per 100,000 live births, while neonatal mortality is estimated at 27 deaths per 1,000 live births [13, 14]. Both rates significantly exceed the Sustainable Development Goals (SDG) targets for reductions in preventable maternal and infant deaths.

Increasing access to ANC will require not only addressing direct financial and geographic barriers, but also addressing the social determinants of health—the “conditions in which people are born, grow, work, live, and age, and the wider set of forces and systems shaping the conditions of daily life” [15]—that influence who is able to access care. Among the social determinants with the greatest potential for impact is mothers’ access to education, as it is associated with increased utilization of ANC. An analysis of ANC access across 32 SSA countries found that 91.3% of women with a secondary education, compared to just 79.4% of those with a primary education and 62.5% with no formal education, received at least some ANC services from skilled providers [8].

Yet little research has rigorously examined whether policies designed to increase girls’ access to education have a direct impact on ANC. At the primary level, a small number of causal studies, mostly focused on single countries, have found that eliminating tuition—thereby removing a barrier to access that disproportionately affects girls—increased the number of ANC visits among pregnant women who were exposed to the tuition-free policies [16–19]. At the secondary level and beyond, existing evidence is sparse and inconsistent when it comes to the impact of education policies on ANC. In Peru, an additional year of maternal education due to the expansion of compulsory schooling from five to 11 years in 1993 increased the likelihood of at least one ANC visit by 1% point [20]. In Bangladesh, an evaluation of a project that made secondary education free for girls residing in rural areas and also provided extra stipends increased the number of ANC visits by 0.34 [21]. In contrast, a study of the educational reforms undertaken in Zimbabwe in 1980—which eliminated primary tuition, established automatic grade progression to secondary, and initiated large-scale construction of secondary schools—found that the reforms increased maternal educational attainment but had no significant effect on ANC [22]. To our knowledge, no study has directly measured the impact of large-scale tuition-free secondary education policies on ANC.

**Fig. 1:**
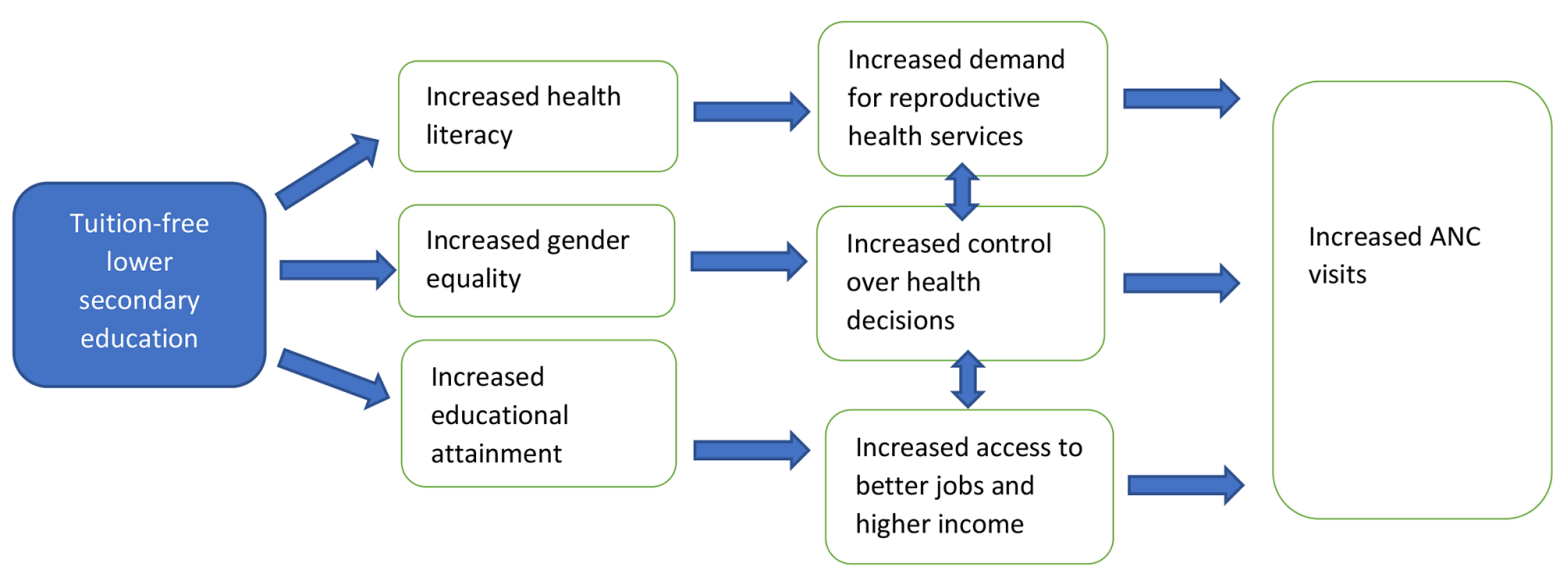
Pathways for tuition-free lower secondary education to increase ANC visits

We hypothesize that increased access to secondary school is likely to have a greater impact on ANC visits by women than primary education. There are three main pathways through which secondary education could increase ANC visits, as presented in Fig. 1. First, access to secondary education is likely to increase health literacy and health-seeking behavior [20–23]. Second, increased educational attainment for women is associated with greater empowerment for women, including more control over personal health decisions [20–22]. Third, higher educational attainment supports access to better and more highly paid jobs, reducing financial barriers to reproductive health care [20–22, 24].

Examining the impacts of tuition-free education on ANC across sub-Saharan African countries, and in particular the impacts of tuition-free secondary education, is an important undertaking as countries seek to implement new strategies to advance their global and regional commitments to both health and education. Through the SDGs, all countries have agreed to not only “ensure universal access to sexual and reproductive health-care services” (SDG 3.7), but also to “ensure that all girls and boys complete free, equitable and quality primary and secondary education” (SDG 4.1). The Maputo Protocol likewise includes strong protections for both reproductive health and equal access to education. Moreover, although most countries in SSA have fully eliminated tuition at the primary level, many still charge tuition to begin or complete secondary schooling, indicating that this is a ripe area for further policy reform. At the same time, while some valuable evidence suggests that tuition-free primary makes a difference for ANC, less is known about tuition-free secondary.

This study fills this research gap by investigating the impact of national-level policies that eliminate tuition fees for lower secondary education in SSA on the number of ANC visits. The number of ANC visits is a critical metric for studying maternal and infant health globally. The WHO recommended a minimum of four ANC visits in 2002 and revised this minimum upwards to eight visits in 2016 [11], while continuing to monitor for four visits. In 2016, the WHO also published a comprehensive set of guidelines on ANC for women and adolescent girls with 39 recommendations related to nutrition, maternal and fetal assessment, preventive measures, interventions for common physiological symptoms, and health system [11]. The number of ANC visits was used as a simple indicator to monitor progress towards the goal of safe reproductive health in the MDGs; today it remains an important metric used by the WHO to measure progress over time and across countries.

Using data from nine SSA countries and employing a quasi-experimental methodology, we provide the first cross-country estimates of the effect of tuition-free lower secondary education on ANC coverage. We also examine whether making lower secondary education free has a greater impact on ANC utilization compared to making primary education free. As countries seek to realize their SDG commitments while also making the most efficient use of limited resources, understanding to what extent policies that move the social determinants of health—including education—are likely to directly advance specific health goals can help inform evidence-based policymaking. The findings of this study can inform policymakers about the potential benefits of tuition-free secondary education in improving ANC coverage and addressing the issue of low utilization.

## Methods

To estimate the effect of women’s exposure to tuition-free education policies at the primary and lower secondary levels on their ANC visits, a difference-in-difference (DD) methodology was employed in this study. The DD approach is a quasi-experimental strategy that enables the comparison of outcomes between women who were exposed to the policy and those who were not, both within treatment countries and in comparison to women in comparison countries during the same time frame. This analysis leverages the variation in the timing of education policies across SSA countries. Specifically, the comparison was made based on whether women were exposed to tuition-free education policies, which was determined by their country of residence and year of birth.

### Data

Country-level data on tuition-free education policies and legislation was obtained from a novel database constructed by the WORLD Policy Analysis Centre. This database contains details of education laws and policies from 1990 to 2019 in all African countries with at least two DHS surveys, including whether education at various levels of schooling was made tuition-free, the years of adoption of the policies, and the official ages of schooling. The database was constructed primarily using national legislation and official country documents accessible via the United Nations Educational, Scientific and Cultural Organization’s (UNESCO) Observatory on the Right to Education. Data was coded independently by two researchers who then reconciled their answers. Country reports and other secondary sources were consulted to verify that constitutional rights, laws, and policies that made education tuition-free were implemented. Countries were coded as not having tuition-free education if it was evident from these sources that tuition fees were still being charged despite legal guarantees. Other forms of fees, such as textbooks or uniforms, were not considered in the analysis.

The individual-level data used in this study comes from the Demographic Health Surveys (DHS).^1^ The DHS are nationally representative cross-sectional household surveys of women aged 15–49 years conducted in low- and middle-income countries. The DHS are well suited for our analysis because they collect detailed information on women’s birth histories, including the number of ANC visits. Our sample includes countries with at least two years of DHS data available since 1999. This cutoff was chosen to ensure that we had information on policy exposure for women born in 1984 or later, considering the availability of policy data from 1990 and the typical age of starting primary school at six years across most African countries. The earliest year in which women born in 1984 can be surveyed is 1999.

Additional country-level data were obtained from the World Development Indicators. These include gross domestic product (GDP) per capita, the share of the population living in urban areas of the country, and the domestic health expenditure as a percentage of GDP. All relevant variables used constant 2015 dollars.^2^

### Outcomes

The main outcome in this study is the number of ANC visits for the most recent birth that occurred within the five years prior to the survey. During the survey, women were asked whether they sought ANC for the pregnancy preceding their most recent live birth, and if so, they were further queried about the frequency of care received. Women who reported uncertainty about the number of ANC visits or reported more than 21 visits were excluded from the analysis. To facilitate comparison with existing literature, we also constructed a binary variable indicating whether a woman had attended a minimum of four ANC visits, as recommended by the WHO at the time of the pregnancy [10].^3^

### Estimation strategy

Using a two-way fixed effects (TWFE) model, we estimated the following equation:

1 $$\begin{gathered}{y_{icbt}} = {\beta _1}Bot{h_{icb}} + {\beta _2}Primar{y_{icb}} + {\gamma _1}ag{e_{ict}} \hfill \\\,\,\,\,\,\,\,\,\,\,\, + Countr{y_c} + Cohor{t_b} + Yea{r_t} + {e_{icbt}} \hfill \\ \end{gathered}$$

where ${y}_{icbt}$ is the outcome for woman $i$ born in year $b$ in country $c$ and surveyed in year $t.$ The indicator ${Both}_{icb}$ takes a value of 1 if woman $i$ born in year $b$ was exposed to both free primary and lower secondary education in country $c.$ Similarly, the indicator $Primar{y}_{icb}$ takes a value of 1 if woman $i$ born in year $b$ was exposed to free primary education in country $c$ but not to lower secondary education. A woman is considered exposed to free primary education if her age at the time of the adoption of the tuition-free primary education policy was no more than two years older than the minimum age of primary school entry in a treatment country. For instance, if the minimum age of entry into primary school is 6 years, any girl who was under the age of 8 at the start of the policy is considered exposed to free primary education. This definition accounts for grade repetition and late entries into school, which are common in Africa [26].^4^

A woman is defined as being exposed to free lower secondary education if she reached the minimum age of entry to lower secondary school in the year of the policy or later in a treatment country. Women who were expected to enter school prior to the year of the education policy were considered never exposed to the policy. Women exposed to free lower secondary but not free primary were excluded from the analysis as they paid the primary school tuition fees and are likely to differ from women with access to free education throughout their school years.

The regression model includes fixed effects for countries ($Countr{y}_{c}$) and birth years ($Cohor{t}_{b}$). The country fixed effects control for time-invariant country characteristics, while the birth year fixed effects control for time-specific factors common to birth cohorts across all countries. Additionally, a set of survey year dummies (${Year}_{t}$) captures changes over time that are common to all countries and birth cohorts. The woman’s age at the time of the survey is also included as a control variable.^5^ The regression accounts for time-varying country characteristics that are correlated with the outcome or the adoption of the education policies, such as domestic health expenditure as a percentage of GDP as well as the share of the urban population at the time of the survey, and GDP per capita at the time of the policy.

The parameters ${\beta }_{1}$ and ${\beta }_{2}$represent the reduced-form or intent-to-treat estimates of the average effects of exposure to tuition-free education at both levels (primary and lower secondary) and tuition-free education at the primary level only, respectively, relative to women unexposed to either policy in the treatment and comparison countries. To assess the additional effect of free lower secondary education over free primary education, a Wald test was conducted to test the difference between ${\beta }_{1}$ and ${\beta }_{2}$.

The regression model was estimated using pooled data from all countries. Additionally, the model was estimated separately for each treatment country while keeping the comparison countries unchanged. This approach allows for the examination of heterogeneous effects of the free education policies across the treatment countries, which may be obscured in the pooled analysis. A Poisson regression model was used when the outcome is a count variable, taking on nonnegative integer values, while a Probit model was used when the outcome is a binary variable. Ordinary least squares regression is not suitable for outcomes with limited ranges for various reasons [27]. Standard errors were clustered at the country level, and the DHS weights were utilized after de-normalization according to the DHS Sampling and Household Listing Manual [28].^6^ All analyses were conducted using STATA 14.2.

### Treatment and comparison countries

To apply the DD methodology, it is necessary to have data on women exposed and unexposed to the tuition-free education policies in countries that adopted such policies (treatment countries) and on women in countries with no similar policies (comparison countries). Since we want to separate the impact of tuition-free lower secondary education policy from the impact of tuition-free primary education policies, only countries with staggered adoption of the two policies can serve as treatment countries. This is because when countries made lower secondary policy free some years after making primary education free, they are likely to contain distinct birth cohorts of women exposed to the free primary education policies only and to both education policies.

The analysis focused on three treatment countries: Liberia, Rwanda, and Zambia. These countries exhibited sufficient policy lags that allowed for the differentiation of women who attended school under the free primary education policy from those who attended school under both the free primary and lower secondary education policies. These were selected because they were the only African countries with policy and outcome data, that (1) introduced tuition free education for all children, (2) staggered their introduction of tuition free primary and secondary so the effects of each could be studied and (3) had sufficient data on the ANC visits of women who had experienced tuition free primary and tuition free secondary. Policy years for all countries are provided in Appendix Table A1.

The countries selected for comparison were those that did not implement any tuition-free education policies during the period under consideration or did not introduce them early enough relative to their most recent DHS surveys. These comparison countries included Democratic Republic of Congo (DRC), Mozambique, Niger, and Zimbabwe. Benin and Burundi, which had free primary education policies, were also treated as comparison countries by excluding the cohorts exposed to the policy in each country. This was not possible with the other African countries with free primary education policies as there were no observations corresponding to the post-intervention years in any of the three treatment countries. The timing of the policy interventions in the treatment and comparison countries is presented in Table 1, which also provides information on the expected age of students at each level and the birth cohorts expected to be exposed to the policies, if at all, in each country within our final sample.

**Table 1:** Policy details of the treatment and comparison countries

	Free primary			Free lower secondary		
	Policy year	Min. school age	Birth year of first exposed cohort	Policy year	Min. school age	Birth year of first exposed cohort
	**Treatment countries**					
Liberia	2006	6	1998	2011	13	1998
Rwanda	2004	7	1995	2009	13	1996
Zambia	2003	7	1994	2011	14	1997
	**Comparison countries**					
Benin	2006	6	1998	.	12	
Burundi	2005	7	1996	.	13	
DRC	2014	6	2006	2014	12	2002
Mozambique	2005	6	1997	.	13	
Niger	.	7		.	13	
Zimbabwe	.	6		.	13	

Notes: Birth year of the first cohort exposed to a free primary education policy = (policy year – (minimum age of entry into primary school + 2)). Birth year of the first cohort exposed to a free lower secondary education policy = (policy year – minimum age of entry into lower secondary). Although the birth cohorts for Liberia are the same, there are cohorts with differential policy exposure due to a change in the expected years of schooling during the time period of interest

### Analytic sample

We constructed a repeated cross-sectional dataset by pooling data from multiple Demographic and Health Surveys (DHS) conducted in nine countries. The dataset included women born between 1984 and 2004 who had given birth to their most recent singleton child within five years of the survey. However, we excluded women born in 1984 and 1985 in Mozambique due to the uncertainty surrounding the education policies implemented in the country in 1990 and 1991. Similarly, women born in 1984 in Zimbabwe were excluded because the country introduced tuition-free primary education in 1990 but reversed the policy the following year.

To ensure the integrity of the data and avoid potential contamination from the COVID-19 pandemic, we excluded women whose most recent child was born in March 2020 or later. Additionally, women with missing outcome values were removed from the analysis. This resulted in a final sample size of 67,738 women. The specific DHS used in each country and the corresponding sample sizes are presented in Table A2.

### Robustness checks

We conducted several checks to verify the robustness of our main findings. First, we re-estimated Eq. (1) using a negative binomial model instead of the Poisson model. The negative binomial model is appropriate when count data exhibits overdispersion, which occurs when the variance exceeds the mean [29]. Although Poisson estimates are consistent even in the presence of non-Poisson distributed outcomes [27, 29], the standard errors may be imprecise.

Second, we restricted our analysis to women born in birth cohorts that were common across both the treatment and comparison countries. This was necessary due to differences in survey timing and education policy implementation. Figure A2 illustrates the birth cohorts for women in the treatment countries, who were born between 1984 and 2004, and women in the comparison countries, who were born between 1984 and 2000.

Third, we limited the age range of women included in the analytical sample to ensure comparability between exposed and unexposed women across all countries. Specifically, we included women aged 15 to 30 years in the sample. While women exposed to free lower secondary education ranged in age from 15 to 24 years, the upper age limit for unexposed women in both the treatment and comparison countries exceeded 30 years.^7^

Fourth, we addressed the potential bias in TWFE when there is staggered implementation of the treatment of interest and the average treatment effects vary over time [30–32]. In such cases, the TWFE estimator may assign negative weights to some treated observations, which can be problematic if treatment effects vary over time. To assess the extent of negative weighting, we examined the proportional relationship between weights and residuals from a regression of treatment on country and year fixed effects [33]. We then dropped the treated country-years with negative weights and re-estimated Eq. (1) to mitigate the issue of biased TWFE estimation.

## Results

### Summary statistics

Individual-level summary statistics are presented in Table 2. The mean number of ANC visits is greater for the women exposed to tuition-free primary and lower secondary education policies in the treatment countries than for women unexposed to the policy in the treatment and comparison countries. The similar pattern is observed for the proportion of women with four or more ANC visits. The mean age of the women exposed to the policies in the treatment countries is 20 years, while the women unexposed to the policies are relatively older, with mean ages of 25 and 24 years in the treatment and comparison countries, respectively.

**Table 2:** Summary statistics

	Treatment		Comparison
	Women unexposed to free education	Women exposed to free education	Women unexposed to free education
Number of ANC visits	4.059	4.112	3.668
	(2.031)	(1.865)	(2.223)
Share of women with at least 4 ANC visits	0.577	0.614	0.509
	(0.494)	(0.487)	(0.500)
Age	25.19	19.59	23.59
	(4.462)	(2.053)	(3.895)
Observations	25,996	3145	38,597

Notes: Treatment countries are Liberia, Rwanda, Zambia; comparison countries are Benin, Burundi, DRC, Mozambique, Niger, Zimbabwe. Free education refers to tuition-free primary and lower secondary education

### Pre-treatment trends

**Fig. 2:**
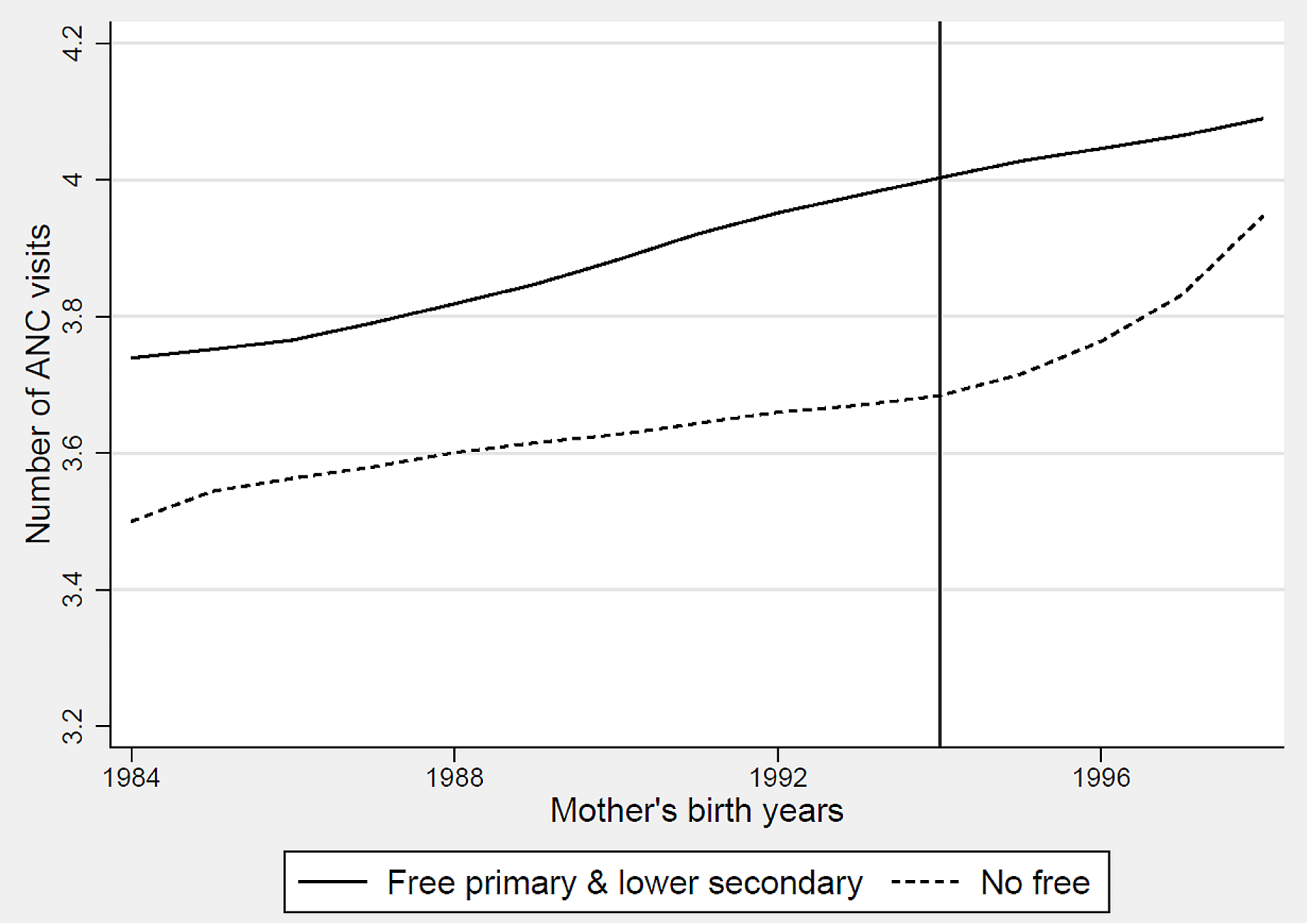
Trends in mean number of ANC visits (with Loess smoother). Notes: Treated countries with free primary and lower secondary education policies are Liberia, Rwanda, Zambia, and comparison countries are Benin, Burundi, DRC, Mozambique, Niger, Zimbabwe. The black vertical line indicates the year 1994 when the earliest birth cohort was exposed to tuition-free primary education among the three treatment countries

Figure 2 depicts the pre-treatment trends in the average number of ANC visits for both the treatment and comparison countries. The graph demonstrates that initially the trends in the treatment and comparison countries evolved similarly. However, around 1994, which corresponds to the earliest birth cohort exposed to tuition-free primary education among the three treatment countries, the outcome in the treatment countries began to deviate from that of the comparison countries. This observation indicates that in the absence of the education policies, the divergence between the treatment and comparison countries would have persisted over time. Consequently, the parallel trends assumption, which is crucial for the causal interpretation of the DD method, was not violated.

We further evaluated the pre-treatment trends in the treatment and comparison countries by estimating a regression model with treatment leads and lags along with country fixed effects, birth year fixed effects, survey years fixed effects, and control variables. The results of this analysis are presented in Figure A2 in the Appendix. The coefficients on the leads, binary variables indicating the years prior to the exposure of women to both tuition-free education policies in a country, are generally not statistically significant, indicating support for the parallel trends assumption. The graph also indicates that the number of ANC visits significantly increase over time following the implementation of the education policies.

### Impact of the free education policies

Table 3 presents the average marginal effects (AME) based on the Poisson regression model for the DD estimates. In Column 1, which includes all treatment and comparison countries, the results indicate a statistically significant positive AME for the $Both$ term, with a magnitude of 0.189. This suggests that women exposed to both the free primary and lower secondary education policies in the treatment countries had, on average, 0.19 more ANC visits compared to women who were not exposed to the policy. In percentage terms, this corresponds to a 5% increase in ANC visits ($Both$/Mean = 0.189/3.799).

Furthermore, the results show that exposure to only the free primary education policies in the treatment countries, as indicated by the estimated coefficient on the $Primary$ term, is associated with 0.09 more ANC visits, which is significantly smaller than the increase observed when women are exposed to both policies.

**Table 3:** AME of free education policies on ANC visits

	(1)	(2)	(3)	(4)
	Pooled	Liberia	Rwanda	Zambia
Exposed to free primary and lower secondary (Both)	0.189**	0.183***	0.323***	0.333***
	(0.095)	(0.043)	(0.061)	(0.087)
Exposed to free primary only (Primary)	0.093*	0.306**	0.116	0.163***
	(0.054)	(0.125)	(0.072)	(0.049)
Observations	67,738	45,376	49,078	50,478
Both – Primary	0.0960	-0.123	0.207	0.170
F-test	3.140	0.712	6.165	7.499
pval	0.0760	0.399	0.0130	0.00600
Mean outcome in pre-policy period	3.799	3.948	3.569	3.703

Notes: Treatment countries are Liberia, Rwanda, Zambia, and comparison countries are Benin, Burundi, DRC, Mozambique, Niger, Zimbabwe. *** *p* < 0.01, ** *p* < 0.05, * *p* < 0.1. Standard errors in parentheses are clustered at the country level. The regressions include country, birth cohort, and survey year fixed effects. We control for mothers’ age, domestic health expenditure as percent of GDP at the time of the survey, share of urban population at the time of the survey, and GDP per capita at the time of the policy. To calculate the mean number of ANC visits for women in the pre-policy period, we used the birth years before the first cohort exposed to both policies was born.

Columns 2 to 4 in Table 3 present the results of each treated country individually. We find that exposure to free primary and lower secondary education is significantly associated with 0.19 to 0.3 more ANC visits in Liberia, Rwanda, and Zambia. This translates to a 5–9% increase in ANC visits among women exposed to the policies compared to the unexposed women. Additionally, we observe that exposure to both policies is associated with significantly more ANC visits than exposure to free primary education only in Rwanda and Zambia. Note that the country-specific estimates are sensitive to the choice of comparison countries and should be interpreted cautiously.

Next, we examine the impact of the policies on the binary outcome of having at least four ANC visits. Table 4 presents the AME for the Probit regression model. While the pooled result for $Both$ is no longer statistically significant, the significant positive association found in Table 3 between the education policies and outcome persists for each treatment country individually. Exposure to the free primary and lower secondary education policies in these three countries is associated with a 6 to 14% higher share of women with four or more ANC visits compared to the unexposed women. Notably, the impact of free lower secondary education is more pronounced in Rwanda and Zambia, while free primary education is more effective in improving ANC coverage in Liberia.

**Table 4:** AME of free education policies on at least 4 ANC visits

	(1)	(2)	(3)	(4)
	Pooled	Liberia	Rwanda	Zambia
Exposed to free primary and lower secondary (Both)	0.028	0.077***	0.031*	0.061***
	(0.019)	(0.011)	(0.017)	(0.015)
Exposed to free primary only (Primary)	0.019**	0.100***	-0.017	0.033***
	(0.010)	(0.007)	(0.012)	(0.008)
Observations	67,730	45,367	49,068	50,478
Both – Primary	0.00900	-0.0230	0.0470	0.0280
F-test	0.481	2.775	7.777	6.874
pval	0.488	0.0960	0.00500	0.00900
Mean outcome in pre-policy period	0.532	0.548	0.492	0.524

Notes: Treatment countries are Liberia, Rwanda, Zambia, and comparison countries are Benin, Burundi, DRC, Mozambique, Niger, Zimbabwe. *** *p* < 0.01, ** *p* < 0.05, * *p* < 0.1. Standard errors in parentheses are clustered at the country level. The regressions include country, birth cohort, and survey year fixed effects. We control for mothers’ age, domestic health expenditure as percent of GDP at the time of the survey, share of urban population at the time of the survey, and GDP per capita at the time of the policy. To calculate the mean share of women with at least 4 ANC visits in the pre-policy period, we used the birth years before the first cohort exposed to both policies was born.

### Sensitivity analysis

Table 5 presents the estimated coefficients from the robustness checks. In Column 1, we replicate our baseline findings from Table 3 for comparison. In Column 2, we report the results from a nominal binomial regression model used to account for the possibility of mis-specifying the distribution of the outcome as a Poisson. The results remain unchanged. Columns 3 and 4 restrict the sample by birth cohorts and ages, respectively, to ensure comparability of the women exposed and unexposed to the policies across all countries. We find that AMEs for $Both$ are positive and statistically significant in both cases, although the magnitudes and precisions of the estimates vary compared to the main results. The results for the treatment country-specific analysis also withstand these checks, and detailed results can be provided upon request.

In Column 5, we drop the last few birth cohorts in Rwanda and Zambia where negative weights were observed, as depicted in Figure A3, which displays the weights assigned to country-year level observations for calculating the TWFE and the distribution of negative weights across country-year observations. Even after this adjustment, we continue to observe a significant and positive association between the tuition-free education policies and the number of ANC visits.

**Table 5:** Sensitivity analyses results

	(1)	(2)	(3)	(4)	(5)
	Main	Negative binomial	Overlapping birth cohorts	Ages 15–30	Excluding country-years with negative weights
Exposed to free primary and lower secondary	0.189**	0.189**	0.211**	0.052**	0.053**
	(0.095)	(0.096)	(0.087)	(0.025)	(0.025)
Exposed to free primary only	0.093*	0.093*	0.103**	0.022	0.026*
	(0.054)	(0.054)	(0.051)	(0.014)	(0.014)
Observations	67,738	67,738	67,229	62,615	67,704

Notes: Treatment countries are Liberia, Rwanda, Zambia, and comparison countries are Benin, Burundi, DRC, Mozambique, Niger, Zimbabwe. *** *p* < 0.01, ** *p* < 0.05, * *p* < 0.1. Standard errors in parentheses are clustered at the country level. The regressions include country, birth cohort, and survey year fixed effects. We control for mothers’ age, domestic health expenditure as percent of GDP at the time of the survey, share of urban population at the time of the survey,  and GDP per capita at the time of the policy.

## Discussion

Adequate access to ANC is fundamental to reproductive health and critical to reducing maternal and infant mortality. Yet half of women in SSA still are not obtaining at least four ANC visits during pregnancy [8, 12]. Alongside improvements in service delivery, closing the gaps in access will require addressing the social determinants of reproductive healthcare access, including education. While maternal education has been linked with receiving ANC, little research has examined whether specific education policy changes can have impact. This is the first study to estimate the impact of national-level tuition-free lower secondary education policies on ANC across countries in SSA, and to assess the relative impacts of policies making primary and lower secondary education free.

The results indicate that exposure to tuition-free primary and lower secondary education is associated with a 5% increase in the average number of ANC visits. Moreover, the impact of both education policies combined is greater than that of tuition-free primary education alone. Exposure to tuition-free primary and lower secondary education policies also increased the share of women meeting the WHO recommendation of four or more ANC visits by 6–14%. However, it should be noted that the impact of these policies varies across the three individual treatment countries, with Liberia showing a greater effect for tuition-free primary education than for both levels. There could be several reasons for the varying effectiveness of tuition-free lower secondary in the different treatment countries. For example, countries may have invested different amounts in school budgets, impacting access to education and the quality of education provided. In some countries, other forms of fees may still persist, such as textbooks or school uniforms, creating barriers to education. Implementation of new tuition-free policies may also vary across countries with some countries being faster or slower to realize the promise of tuition-free lower secondary education for all students. Further research is necessary to understand the dynamics in operation in each treatment country, including the role of implementation efforts. The country-specific results are supported when we look at whether women had at least four ANC visits.

The study contributes to the existing literature on the elimination of school fees at the secondary level, which has demonstrated positive effects on a range of important outcomes including education expenditures, enrollment rates, test scores, and labor market outcomes [34]. Our findings align with previous research highlighting the broader benefits of reducing financial barriers to secondary schooling to areas that matter to reproductive health such as fertility, age at first childbirth, and health behaviors [35, 36]. This study also adds value to two studies that have demonstrated that tuition-free lower secondary matters more than tuition-free primary. Bhuwania and Heymann [37] demonstrated that making lower secondary education tuition-free improves women’s attitudes towards intimate partner violence being unjustified, which are in turn linked to higher access to ANC [38–40]. Tuition-free lower secondary has also been demonstrated to matter more than tuition-free primary in reducing the incidence of early childbearing, a risk factor for maternal mortality [41].

With policymakers, intergovernmental organizations, and other stakeholders seeking to identify effective and efficient policy levers for improving ANC coverage, this article’s findings demonstrate that removing the tuition barrier can meaningfully advance both reproductive health in addition to greater educational attainment, with the full range of its attendant benefits. It is important to recognize that while tuition-free primary education has been widely implemented across Africa, a significant number of countries still charge tuition fees at the secondary level [42]. As governments across the continent turn their attention to eliminating tuition at the secondary level, this study offers new evidence about its potential impacts on reproductive health outcomes of women and adolescents. While expanding access to free education requires investment, the long-term human and economic benefits will substantially outweigh the costs. Moreover, in the short-term, global funds have a role to play in ensuring the provision of tuition-free education is affordable for countries at all income levels. In the long-term, as greater educational attainment grows countries’ economies, they will be able to meet the full costs themselves.

Several limitations of the study should be acknowledged. Firstly, the study relies on self-reported data from the DHS, which may be subject to recall bias. However, this bias is minimized since we focus on the most recent live birth within a limited time frame. Secondly, the analysis does not account for treatment-on-the-treated effects due to the unavailability of DHS data on the timing of women’s school entry, grade repetition, where she went to school, and whether she migrated from the area. Consequently, the estimates assume that women entered school within two years after the expected minimum age for primary education and remained in the same country at the time of the survey.

Third, the TWFE may be biased when there are staggered treatments with heterogenous effects over time [30–32] or multiple treatments [43]. Although we found few treatment country-year observations receive negative weights when testing for the extent of negative weighting and we conducted a simple robustness check by excluding country-year observations with the negative weights, the TWFE estimates should be treated with caution in the presence of potential heterogeneous treatment effects [33]. Fourth, the validity of the DD design is subject to potential confounding factors, such as the introduction of other sub-national or national policies and programs affecting the outcome during the study period.

Lastly, it is important to acknowledge that the elimination of tuition fees is only one aspect of addressing barriers to girls’ educational attainment. Other factors, such as school supplies, uniforms, sanitation facilities, and safe transportation, can also impede access to education. However, the abolishment of tuition fees is an internationally agreed upon standard and for many countries represents an important milestone towards increasing enrolment for girls. Moreover, some countries impose tuition fees in upper secondary. Future studies should consider examining the impact of broader interventions alongside policies making education tuition-free through the completion of secondary to provide a more comprehensive understanding of the factors influencing girls’ educational attainment.

Despite these limitations, this study highlights the positive consequences of scaling up policy investments in secondary education for ANC coverage. By eliminating tuition fees for secondary school, countries can make significant strides towards improving educational attainment and ultimately achieve better antenatal coverage, contributing to the attainment of the SDGs related to maternal and infant health. Given the substantial human and economic costs of inaction, investing in girls’ education emerges as a powerful strategy for enhancing antenatal care outcomes.

### Electronic supplementary material

Below is the link to the electronic supplementary material.

Supplementary Material 1

## Data Availability

All data are publicly available from https://dhsprogram.com/.

## Footnotes

1 Data for most countries was obtained from the IPUM-DHS [25]. When a survey was not available on IPUMS-DHS, it was harmonized by the staff at the WORLD Policy Analysis Centre using the IPUMS codebook to ensure consistency of methods.

2 We imputed the values of the country-level indicators when they were missing.

3 We do not use an outcome based on the 2016 WHO recommendation of at least 8 ANC visits because the DHS and hence the births in some of the countries occurred before 2016.

4 The UNESCO report on grade repetition suggests that repetition accounted for more than 1 year of the school life expectancy in 2000 and 2010 in Sub-Saharan Africa.

5 We do not control for other individual-level variables since they are likely to mediate the association between the dependent and independent variables, such as rural-urban, household size, years of education, etc.

6 The standard weights available in the DHS data are relative weights which are normalized so that the total number of weighted cases is equal to the total number of unweighted cases. Using the standard DHS weights for pooled data analysis implies giving more weight to surveys with more data. Therefore, the DHS recommends de-normalizing the standard weights for pooled data analysis, that is, multiply the standard weights by the target population and divide by the number of completed cases, for each survey.

7 Inclusion of women aged 25 to 30 years allows us to compare children of exposed and unexposed mothers within the same country in the most recent surveys.
