# Supplementary material for: Do tuition-free lower secondary education policies matter for antenatal care among women in sub-saharan African countries?
Source: BMC Pregnancy Childbirth. 2024 Apr 8;24:250. doi: 10.1186/s12884-024-06406-1 (PMC11000362; doi:10.1186/s12884-024-06406-1)
Supplement: Supplementary file 1 — Supplementary Material 1 [file 12884_2024_6406_MOESM1_ESM.docx]

**Do Tuition-Free Lower Secondary Education Policies Matter for Antenatal Care among Women in Sub-Saharan African Countries?**

**Appendix**

**Table A1: Policy details of potential countries**

| **Countries** | **Year of tuition-free primary education policy** | **Year of tuition-free lower secondary education policy** |
| --- | --- | --- |
| Benin | 2006 | . |
| Burkina Faso | 2007 | 2007 |
| Burundi | 2005 | . |
| Cameroon | 2000 | . |
| Chad | 2006 | 2006 |
| Congo, Republic of | 1995 | 1995 |
| DRC | 2014 | 2014 |
| Egypt | 1981 | 1981 |
| Ethiopia | 1995 | 1995 |
| Gabon | 1966 | 1966 |
| Gambia* | 1998 | 2004 |
| Ghana | 1961 | 1961 |
| Kenya* | 2003 | 2008 |
| Lesotho | 2000 | . |
| Liberia* | 2006 | 2011 |
| Madagascar | 2002 | . |
| Malawi | 1995 | 1995 |
| Mali | 1962 | 1962 |
| Mozambique | 2005 | . |
| Namibia* | 2001 | 2016 |
| Niger | . | . |
| Rwanda* | 2004 | 2009 |
| Senegal | 2004 | 2004 |
| Sierra Leone* | 2000 | 2004 |
| Tanzania* | 2002 | 2016 |
| Uganda* | 1997 | 2007 |
| Zambia* | 2003 | 2011 |
| Zimbabwe | . | . |

Note: * indicates countries with staggered adoption of the two policies. Namibia and Tanzania were excluded from the analysis due to the lack of data on girls influenced by the lower secondary education policy in their most recent Demographic Health Surveys (DHS), as these policies are relatively new in these countries. Kenya and Sierra Leone had such a short gap between the two policies that there was an overlap between birth cohorts exposed to the primary education policy and those exposed to both policies. In Gambia, there was no age overlap between women with no exposure to free education policies and those exposed to tuition-free primary and lower secondary education policies. Additionally, Uganda had a unique free education policy that applied only to households with four or more children.

**Figure A1: Treatment timing plots**

|  | **Birth years of women** | | | | | | | | | | | | | | | | | | | | |
| --- | --- | --- | --- | --- | --- | --- | --- | --- | --- | --- | --- | --- | --- | --- | --- | --- | --- | --- | --- | --- | --- |
|  | **1984** | **1985** | **1986** | **1987** | **1988** | **1989** | **1990** | **1991** | **1992** | **1993** | **1994** | **1995** | **1996** | **1997** | **1998** | **1999** | **2000** | **2001** | **2002** | **2003** | **2004** |
|  | **Final set of treatment & comparison countries** | | | | | | | | | | | | | | | | | | | | |
| Liberia |  |  |  |  |  |  |  |  |  |  |  |  |  |  |  |  |  |  |  |  |  |
| Rwanda |  |  |  |  |  |  |  |  |  |  |  |  |  |  |  |  |  |  |  |  |  |
| Zambia |  |  |  |  |  |  |  |  |  |  |  |  |  |  |  |  |  |  |  |  |  |
| DRC |  |  |  |  |  |  |  |  |  |  |  |  |  |  |  |  |  |  |  |  |  |
| Mozambique |  |  |  |  |  |  |  |  |  |  |  |  |  |  |  |  |  |  |  |  |  |
| Niger |  |  |  |  |  |  |  |  |  |  |  |  |  |  |  |  |  |  |  |  |  |
| Zimbabwe |  |  |  |  |  |  |  |  |  |  |  |  |  |  |  |  |  |  |  |  |  |
| Benin |  |  |  |  |  |  |  |  |  |  |  |  |  |  |  |  |  |  |  |  |  |
| Burundi |  |  |  |  |  |  |  |  |  |  |  |  |  |  |  |  |  |  |  |  |  |
|  | **Potential comparison countries** | | | | | | | | | | | | | | | | | | | | |
| Kenya |  |  |  |  |  |  |  |  |  |  |  |  |  |  |  |  |  |  |  |  |  |
| Senegal |  |  |  |  |  |  |  |  |  |  |  |  |  |  |  |  |  |  |  |  |  |
| Sierra Leone |  |  |  |  |  |  |  |  |  |  |  |  |  |  |  |  |  |  |  |  |  |
| Burkina Faso |  |  |  |  |  |  |  |  |  |  |  |  |  |  |  |  |  |  |  |  |  |
| Chad |  |  |  |  |  |  |  |  |  |  |  |  |  |  |  |  |  |  |  |  |  |
| Madagascar |  |  |  |  |  |  |  |  |  |  | * |  |  |  |  |  |  |  |  |  |  |
| Cameroon |  |  |  |  |  |  |  |  |  |  |  |  |  |  |  |  |  |  |  |  |  |
| Lesotho |  |  |  |  |  |  |  |  |  |  |  |  |  |  |  |  |  |  |  |  |  |
| Namibia |  |  |  |  |  |  |  |  |  |  |  |  |  |  |  |  |  |  |  |  |  |
| Tanzania |  |  |  |  |  |  |  |  |  |  |  |  |  |  |  |  |  |  |  |  |  |

Note: Yellow indicates birth cohorts of women with no exposure to any policy, blue indicates exposure to free primary education only, green indicates exposure free primary and lower secondary education, and red indicates exposure to free secondary education policy only. The bold line in the lower panel indicates the earliest year with women exposed to an education policy in the treatment countries. * indicates that sample size of women born in 1994 in Madagascar is 3, making it too small for inclusion in analysis. We do not include countries with free education policies in 1995 and earlier as potential comparison countries because all women in these countries are exposed to one or both education policies.

**Table A2: DHS surveys in the final treatment and comparison countries**

| **Countries** | **DHS surveys** | **Number of women unexposed to the policy** | **Number of women exposed to the policy** |
| --- | --- | --- | --- |
| Benin | 2001, 2006, 2011-12, 2017-18 | 6086 |  |
| Burundi | 2010, 2016-17 | 9521 |  |
| DRC | 2007, 2013-14 | 10389 |  |
| Liberia | 2007, 2013, 2019-20 | 693 | 960 |
| Mozambique | 2003, 2011 | 1492 |  |
| Niger | 2006, 2012 | 11195 |  |
| Rwanda | 2000, 2005, 2008, 2010, 2014-15, 2019-20 | 6566 | 7591 |
| Zambia | 2001-02, 2007, 2013-14, 2018 | 3176 | 4832 |
| Zimbabwe | 2005-06, 2010-11, 2015 | 5237 |  |

**Figure A2: Event study**

Notes: Treatment countries are Liberia, Rwanda, Zambia, and comparison countries are Benin, Burundi, DRC, Mozambique, Niger, Zimbabwe. We combined leads and lags greater than 8 into one variable each. The graph shows AME from a Poisson regression of the number of ANC visits on leads, lags, country fixed effects, birth year fixed effects, survey year fixed effects and control variables, with 90 % confidence intervals.

**Figure A3: Two-Way Fixed Effects Weights**

1. **Two-Way Fixed Effects Weights, by Treatment Status**

1. **Weights Used in Two-Way Fixed Effects, by Country and Year**
